# Microbiome-based probiotic intervention to reduce chemotherapy-induced gastrointestinal toxicity

**DOI:** 10.3389/fcimb.2026.1887323

**Published:** 2026-09-29

**Authors:** Haoyu Li, Chunguang Hou, Yan Pan

**Affiliations:** 1 Academy of Art and Design, Hankou University, Wuhan, Hubei, China; 2 General Surgery Department, Huangshi Hospital of Traditional Chinese Medicine, Huangshi, Hubei, China; 3 College of Physical Education, Hubei Normal University, Huangshi, Hubei, China

**Keywords:** chemotherapy, gastrointestinal toxicity, gut microbiota, microbial diversity, probiotics, treatment response

## Abstract

**Introduction:**

Chemotherapy is one of the primary treatment options for cancer; however, its gastrointestinal toxicity and disruption of gut microbial homeostasis can adversely affect patient health and treatment outcomes. This study investigated chemotherapy-induced alterations in the gut microbiome and evaluated the effects of probiotic supplementation on microbial diversity, gastrointestinal toxicity, and treatment response in cancer patients.

**Methods:**

The probiotic strain was isolated from a commercial formulation and identified as Lactobacillus casei using biochemical characterization and 16S rRNA gene sequencing. Its secreted metabolites were characterized using Fourier-transform infrared spectroscopy (FTIR), gas chromatography–mass spectrometry (GC-MS), high-performance liquid chromatography (HPLC), and nuclear magnetic resonance (NMR) spectroscopy. A total of 150 patients receiving platinum-based chemotherapy were enrolled and divided into control and probiotic intervention groups. Fecal samples were collected before and after chemotherapy and subjected to 16S rRNA sequencing to assess microbial diversity and taxonomic composition.

**Results:**

Chemotherapy induced significant gut microbial dysbiosis, characterized by reduced alpha diversity, decreased abundance of beneficial bacteria, including Lactobacillus, Bifidobacterium, and Faecalibacterium, and increased abundance of opportunistic pathogens such as Escherichia-Shigella and Klebsiella. In contrast, probiotic-treated patients maintained a relatively balanced gut microbiota and exhibited less pronounced dysbiosis after chemotherapy. Gastrointestinal complications were also reduced following probiotic supplementation. Overall gastrointestinal toxicity was observed in 85% of control patients compared with 65% of probiotic-treated patients, while diarrhea occurred in 60% and 35%, respectively. Grade 3–4 gastrointestinal toxicity was reported in 40% of control patients compared with 20% of probiotic-treated patients. Probiotic supplementation was additionally associated with lower incidence and severity of mucositis, nausea, and vomiting. Chemotherapy responders exhibited a more favorable microbial profile with greater abundance of beneficial commensal bacteria than non-responders. Receiver operating characteristic analysis identified microbial taxa as potential predictors of chemotherapy outcomes, with AUC values of 0.81 for Bacteroides ovatus, 0.78 for Faecalibacterium prausnitzii, and 0.86 for the combined response model. The combined microbial model achieved an AUC of 0.88 for predicting severe gastrointestinal toxicity.

**Discussion:**

Overall, probiotic supplementation may help preserve intestinal microbial homeostasis, reduce chemotherapy-associated gastrointestinal toxicity, and support improved treatment outcomes in cancer patients.

## 1 Introduction

Cancer is one of the main causes of morbidity and mortality around the world and chemotherapy is one of the pillars of treatment of different types of cancer. While the prognosis for chemotherapy-induced survival has improved, chemotherapy is often well-known for the level of side effects it has, especially gastrointestinal toxicity (Anand et al., 2023). Some of the common problems like diarrhea, stomatitis, nausea, vomiting and abdominal discomfort are not only compromising the quality of life of patients but also are associated with interruption of treatment, dose reduction and poor therapeutic outcome. These negative effects are now known to correlate closely with the changes in the gut flora that occur in response to chemotherapy drugs (Akbarali et al., 2022).

The gastrointestinal tract is a unique and complex ecosystem of microorganisms that contributes significantly to three of the four physiological systems intestinal homeostasis, immune regulation, and nutrient metabolism and protection from pathogenic microorganisms in the human body (Paul et al., 2025; Afzaal et al., 2022). A healthy gut flora helps to maintain the integrity of gut barriers and regulate local and systemic immune functions. Chemotherapy, however, can also alter this balance of microbes, causing a dysbiosis with a decrease in microbial diversity, loss of beneficial commensal bacteria, and an increase in opportunistic pathogens (Jiang et al., 2025; Sadeghloo and Sadeghi, 2025; Nazeer et al., 2025). These microbial changes have been linked to inflammation, higher permeability to the gut and gut toxicity.

The reciprocal relationship between the gut microbiota and cancer treatment has been emphasized in recent research. In addition to playing a role in drug toxicity, gut microbiota is also known to modulate host immune responses, drug metabolism and inflammatory signaling pathways, thus impacting the effectiveness of chemotherapy therapy (Guevara-Ramírez et al., 2024). Some beneficial microbial taxa have been correlated with better treatment responses, while others have been correlated with poor therapeutic responses and toxicity (Susiriwatananont et al., 2025). Hence, gut microbial profiles can be used to gain insights into prediction of chemotherapy response and adverse effects.

Probiotics are strains of live microorganisms that benefit the health of humans when given in sufficient quantities; during chemotherapy, probiotics are an interesting approach to reestablish the microbial balance and maintain intestinal health (Latif et al., 2023). Various genera of probiotics such as Lactobacillus and Bifidobacterium species have been shown to possess several benefits for intestinal health including the ability to improve intestinal barrier function, to generate SCFAs, inhibit growth of pathogenic bacteria and to modulate inflammatory response (Vlasova et al., 2016; Matera, 2024). There are several experimental and clinical studies which indicate that the use of probiotics has the potential to decrease gastrointestinal side effects of chemotherapy and treatment tolerability. The exact correlation between the probiotic intervention and modulation of gut microbiota, response to chemotherapy and toxicity, however, is still not completely understood (Rodriguez-Arrastia et al., 2021; Ding et al., 2026; Ahmad et al., 2025; Khorashadizadeh et al., 2024).

Lactobacillus spp. are probiotics that are well known in gastrointestinal health promotion and modulation of host immunity. In addition to their colonization ability, these microorganisms display a spectrum of beneficial properties due to their production and secretion of a variety of bioactive metabolites (organic acids, fatty acid derivatives, peptides, exopolysaccharides and other fermentation derived compounds). These metabolites play a role in inhibiting pathogenic microorganisms, maintaining gut microbial balance, promoting intestinal barrier integrity and regulating inflammatory responses.

In the Previous experimental studies have provided evidence that probiotic interventions can modulate chemotherapy-associated gut dysbiosis and gastrointestinal injury. Yue et al. demonstrated beneficial effects of Bacillus treatment on diarrhea and microbiome restoration in piglets, while subsequent work evaluated a three-Lactobacillus preparation in cyclophosphamide-treated rats. These studies provide important preclinical evidence; however, their findings cannot be directly extrapolated to patients receiving chemotherapy. The present study therefore investigated probiotic-associated changes in gut microbiota and gastrointestinal toxicity in human cancer patients undergoing chemotherapy, while additionally examining treatment response and candidate microbial biomarkers.

In recent years, studies on the characterization of probiotic secretomes have emerged as an exciting field for the understanding of the molecular mechanisms associated with the therapeutic properties of probiotics. Various advanced analytical methods including Fourier Transform Infrared (FTIR) spectroscopy, Gas Chromatography–Mass Spectrometry (GC-MS), High-Performance Liquid Chromatography (HPLC) and Nuclear Magnetic Resonance (NMR) spectroscopy have been widely used in the identification and structural characterization of microbial metabolites. Hence, in addition to its effect on the composition of gut microbiota and chemotherapy-induced GI toxicity, the present study explored the extracellular metabolites produced by the isolated strain of Lactobacillus casei from the commercial probiotic formulation using FTIR, GC-MS, HPLC and NMR analysis, to gain mechanistic insight into its probiotic activity.

The purpose of this study was to examine the changes that occur in the gut microbiota after chemotherapeutical treatment and to assess the effect of probiotic administration on gut microbial diversity, gastrointestinal toxicity and treatment response in cancer patients Colorectal cancer – 38%, Gastric cancer – 32%, Other solid tumors – 30%). In addition, the aim of this study was to find specific microbial signatures related to the outcome of chemotherapy and to investigate the potential of these signatures as new predictive biomarkers of therapeutic efficacy and toxicity. The results of this research can be used to develop new support strategies and personalized treatments based on the microbiome in cancer treatment.

## 2 Materials and methods

### 2.1 Study design and participants

Patients with a diagnosis of cancer who were to undergo chemotherapy treatment (paclitaxel 175 mg/m² (day 1), and cisplatin 25 mg/m² (days 1–3). were prospectively enrolled in this study at Hankou College from 2024-2025. Those who had histologically confirmed malignancy and were undergoing chemotherapy as usual were included. The patients who were included in the study had no previous history of antibiotic, probiotic or prebiotic use within 1 month of sample collection and were age ≥ 18 years. Patients with other chronic inflammatory and gastrointestinal disease, severe co-morbidities were excluded. Participants were excluded if they had used antibiotics, probiotics, or prebiotics within one month before sample collection. No dietary restriction was imposed, and participants continued their usual dietary habits. Because detailed dietary intake was not systematically quantified, dietary variation was considered a potential confounding factor and is acknowledged as a limitation.

Before participation, all participants signed informed consent forms. The study protocol was approved by the Institutional Ethics Committee and performed as per Indian Council of Medical Research (ICMR) guideline for biomedical research on Human participants.

### 2.2 Chemotherapy treatment protocol

Chemotherapy was administered every three weeks as per standard clinical practice guidelines that were based on cancer type and stage. The chemotherapy was TP therapy, which was given in cycles consisting of paclitaxel 175 mg/m² (day 1), and cisplatin 25 mg/m² (days 1–3). Modifications of doses were carried out as appropriate, based on patient tolerability and on adverse effects noted (Biswas et al., 2025). The clinical data such as age, gender, stage of cancer, chemotherapy regimen, and adverse events related to chemotherapy were collected from patients’ medical records during the study period. The efficacy of treatment was assessed 6 weeks following the beginning of chemotherapy, by the Response Evaluation Criteria in Solid Tumors (RECIST version 1.1). Gastrointestinal toxicity assesses during chemotherapy were evaluated and scored using the National Cancer Institute Common Terminology Criteria for Adverse Events (NCI-CTCAE).

### 2.3 Probiotic intervention and administration

The patients in the intervention group were given a specific probiotic in combination with regular chemotherapy. The probiotic intervention consisted of *Lactobacillus casei* and *Bifidobacterium* spp., supplied by Unique Biotech Limited, Hyderabad, India, as a commercial probiotic formulation. Each dose contained approximately 1 × 10¹ CFU of viable probiotic bacteria. The probiotic was administered orally once daily, beginning at the initiation of chemotherapy, and continued throughout the chemotherapy treatment period for approximately 6 weeks. The dosing schedule and duration were kept consistent among participants in the probiotic group. The probiotic preparation was [strain(s) as *Lactobacillus* spp.*, Bifidobacterium* spp.*]*, in a total dose of [1 × 10¹ CFU/day]. The formulation was given orally as capsules/sachets/liquid, from the start of chemotherapy and given during the treatment period (Yang et al., 2025).

The dose and length of time that the probiotics were given were the same for everyone in the intervention group. Patients were advised to continue their supplementation program and not use any other probiotics or antibiotics unless clinically necessary during the study. Compliance was assessed using self-report and follow-up. Throughout the study period, all adverse events associated with probiotic use were reported.

#### 2.3.1 Isolation of probiotic bacteria

To prepare the commercial probiotic formulation, it was aseptically opened and the contents were suspended in sterile physiological saline (0.85% NaCl). These were subjected to serial dilutions and plated on de Man, Rogosa and Sharpe (MRS) Agar plates which is a selective medium for the growth of lactic acid bacteria. The inoculated plates were subsequently incubated at 37 °C for 24-48h under microaerophilic conditions. After the incubation, typical Lactobacillus colonies that were well isolated with creamy white, circular, smooth convex colonies were selected and re-streaked on fresh MRS agar plates for purity. Subsequent morphological and biochemical characterizations were done using pure cultures that were preserved on MRS agar slants at 4 °C.

#### 2.3.2 Biochemical identification of Lactobacillus casei and 16S rRNA sequencing

Biochemical identification of Lactobacillus casei and 16S rRNA sequencing were performed. Biochemical Identification of Lactobacillus casei and 16S rRNA Sequencing were carried out.

The purified bacterial isolate isolated from the commercial probiotic formulation has been morphologically and biochemically characterized. The cell morphology and Gram reaction were determined by Gram staining. The amount of catalase activity was determined with 3% hydrogen peroxide. Other biochemical tests such as carbohydrate fermentation profiles and other standard tests for lactic acid bacteria were performed following the microbiological method. The isolate was preliminary identified as Lactobacillus casei based on the phenotypic and biochemical characteristics.

To confirm the molecular level, the purified culture was subjected to a molecular confirmation by using bacterial genomic DNA extraction kit as per manufacturer protocol. Universal bacterial primers 27F (5′-AGAGTTTGATCCTGGCTCAG3′) and 1492R (5′GGTTACCTTGTTACGACTT-3′) were used to amplify the 16S rRNA gene. The polymerase chain reaction (PCR) amplification was carried out under the standard PCR conditions and the amplified products were confirmed by agarose gel electrophoresis. The purified PCR products were sent out for sequencing. The retrieved sequence was then compared to the sequences found in NCBI GenBank database using BLAST algorithm. The highest sequence similarity to authenticated reference strains indicated the isolate to be Lactobacillus casei.

#### 2.3.3 Preparation of cell-free supernatant

A pure culture of Lactobacillus casei was inoculated into sterile de Man, Rogosa and Sharpe (MRS) broth and allowed to grow under microaerophilic condition at 37 °C for 24–48 hours. After the incubation, the bacterial culture was centrifuged at 8,000 rpm for 15 min at 4 °C to remove the bacterial cells from the culture medium. The supernatant was carefully collected and filtered through sterile 0.22 μm membrane filter to remove any bacterial cells and debris. The resulting sterile cell-free supernatant (CFS) was preserved at 4 °C for short-term analyses or at −20 °C until further analysis by FTIR, HPLC, GC-MS/LC-MS and NMR.

#### 2.3.4 FTIR analysis

The functional groups present in the cell-free supernatant of Lactobacillus casei was analyzed by Fourier Transform Infrared (FTIR) spectroscopy. The lyophilized supernatant sample was combined with KBr (spectroscopic grade) and pressed into pellets. FTIR spectra were obtained on FTIR spectrophotometer within the spectral range 4000–400 cm^−^¹ at 4 cm^−^¹ resolution. The spectra obtained were scanned to check the presence of the bioactive metabolites in the bacterial supernatant with the characteristic nature of functional groups.

#### 2.3.5 HPLC analysis

High-Performance Liquid Chromatography (HPLC) was used to measure the concentrations of the major organic acids and metabolites found in the cell-free supernatant. The filtered supernatant was subjected to analysis after passing through 0.22 μm membrane filter. The separation was performed on a C18 reverse-phase column at ambient temperature. The mobile phase used was acidified water and acetonitrile under optimized conditions at a flow rate of 1.0 mL/min and metabolites were detected by UV detection with identification by comparison with authentic standards.

#### 2.3.6 GC-MS analysis

Volatile and semi-volatile metabolites were identified using Gas Chromatography–Mass Spectrometry (GC-MS) in the cell-free supernatant of Lactobacillus casei. The sample was extracted with suitable organic solvent and then concentrated before the analysis. The GC-MS analysis was carried out on a helium flow capillary column at a fixed flow rate. An optimized oven temperature program was used for metabolite separation. Mass spectrum was obtained in electron ionization mode, and the identification of the compound was done by comparing the mass spectra obtained for the compound with available NIST library mass spectra.

#### 2.3.7 NMR analysis

A structural confirmation of the major metabolites of the bacterial supernatant was performed by Nuclear Magnetic Resonance (NMR) spectroscopy. The purified fraction of metabolites was dissolved in deuterated solvent and loaded into NMR tubes. Proton (¹H) NMR and carbon (¹³C) NMR spectra were obtained on an NMR spectrometer at the correct frequency. Chemical shifts are quoted in parts per million (ppm). Metabolites were identified through their structural features, which were confirmed by interpretation of their spectra.

### 2.4 Sample collection and processing

Fecal samples were collected from all participants within three days prior to chemotherapy initiation (baseline) and again six weeks after treatment commencement (post-treatment). Approximately 50–100 mg of fecal sample was self-collected by each participant using an MGIEasy collection tube and immediately stored at −80 °C until further processing and analysis (Vernaci et al., 2023). Fecal samples were collected by participants using sterile, pre-labelled collection containers supplied by the study team. Participants received standardized instructions regarding sample collection and handling. Samples were transported to the laboratory within the specified collection period and processed under appropriate laboratory conditions. Fecal samples were subsequently stored at −80 °C until DNA extraction. The maximum storage duration was approximately 30 days under refrigerated conditions (4 °C).

Genomic DNA was extracted from fecal samples using a commercially available DNA extraction kit according to the manufacturer’s instructions. The quality and concentration of the extracted DNA were assessed using Nano drop analysis and agarose gel electrophoresis. The extracted DNA samples were stored at −20 °C and used for subsequent microbiome analysis.

### 2.5 Microbiome analysis (16S rRNA sequencing)

Microbial community profiling was performed using 16S rRNA gene sequencing. The extracted genomic DNA was subjected to polymerase chain reaction (PCR) amplification targeting the V3–V4 hypervariable regions of the 16S rRNA gene using specific primers (Zemb et al., 2020). The amplified products were cleaned up and prepared into libraries. Standard Illumina protocols were used to sequence on the Illumina platform using paired end reads. Raw sequencing data was quality filtered to get rid of low-quality reads and chimeric sequences. The sequences obtained through the filtration were grouped into operational taxonomic units (OTUs) with 97% similarity using suitable bioinformatics software.

Taxonomic classification was done using a reference database, e.g., Greengenes/SILVA (Cameron et al., 2021). Each sample’s microbial diversity and composition was evaluated using alpha diversity index (Shannon index) and beta diversity analyses. Differential abundance analysis was used to determine significant differences in microbial taxa between groups.

### 2.6 Assessment of gastrointestinal toxicity

All patients received a full evaluation of gastrointestinal toxicity during the course of the chemotherapy cycles. The incidence of clinical symptoms such as diarrhea, nausea, vomiting, abdominal pain, and mucositis were documented during each treatment visit. The National Cancer Institute Common Terminology Criteria for Adverse Events (NCI-CTC) were used to classify adverse events. In order to analyze patients were classified according to toxicity grades as mild to moderate (Grade 1–2) and severe (Grade 3–4) (Spampinato et al., 2025).

The incidence, duration and severity of gastrointestinal toxicities were evaluated between control and probiotic intervention groups. Any complications to the treatment or dose adjustment because of toxicity were also noted.

### 2.7 Statistical analysis

Statistical analyses were performed using R software (version 4.5.1). For continuous variables, the mean ± standard deviation (SD) was used as appropriate. Frequencies and percentages were used to report categorical variables. Two groups were compared through the use of Student’s t-test and multiple group comparisons were made using one-way analysis of variance (ANOVA). Appropriate statistical tests were performed on the diversity indices of the microbiomes and analysis of similarity (ANOSIM) was used to evaluate differences in microbial composition. Using receiver operating characteristic (ROC) curve analysis, the ability of specific microbial taxa to predict response to and toxicity of chemotherapy was assessed. P value < 0.05 was considered statistically significant.

## 3 Results

### 3.1 Isolation of probiotic bacteria and molecular identification of Lactobacillus casei

Lactic acid bacteria were isolated using the commercial probiotic formulation being cultured on de Man, Rogosa and Sharpe (MRS) agar medium. After 24–48 hours of incubation under microaerophilic conditions at 37 °C, distinct colonies with characteristic morphology of lactic acid bacteria were found. Creamy-white, circular, smooth and convex colonies were selected and purified from recovered colonies by repeated streaking on fresh MRS agar plates.

The purified isolate was then morphologically and biochemically characterized. The isolate showed gram-positive rod-shaped bacterial cells; the Gram stain and catalase test confirmed the presence of lactic acid bacteria. Other biochemical tests such as carbohydrate fermentation assays were done following standard microbiological methods (Carmo de Carvalho e Martins et al., 2022; Amar et al., 2019). The isolate was a typical homofermentative lactic acid bacteria, which is a bacteria that makes acid from carbohydrates that can be fermented but does not show any gas production. In addition, there was a significant reduction in pH in MRS broth when grown, suggesting active production of lactic acid. Based on its morphological and biochemical properties, the isolate was characterized as *Lactobacillus casei*. The cell-free supernatant and metabolite characterization studies were carried out using this isolate. The characteristic is outlined in **Table 1**.

**Table 1:** Baseline demographic and clinical characteristics of patients in the chemotherapy and probiotic groups.

Characteristic	Chemotherapy group (n=75)	Probiotic group (n=75)	P value
Age (years), mean ± SD	57.1 ± 10.2	56.5 ± 10.6	0.72
Male, n (%)	44 (58.7)	44 (58.7)	1.00
Female, n (%)	31 (41.3)	31 (41.3)	1.00
Colorectal cancer, n (%)	29 (38.7)	28 (37.3)	0.87
Gastric cancer, n (%)	24 (32.0)	24 (32.0)	1.00
Other solid tumors, n (%)	22 (29.3)	23 (30.7)	0.86
Locally advanced disease, n (%)	48 (64.0)	48 (64.0)	1.00
Metastatic disease, n (%)	27 (36.0)	27 (36.0)	1.00
Platinum-based chemotherapy, n (%)	75 (100)	75 (100)	1.00
Chemotherapy cycles, mean ± SD	2.8 ± 0.7	2.9 ± 0.6	0.34
Baseline GI toxicity Grade 1–2, n (%)	8 (10.7)	7 (9.3)	0.78
Baseline GI toxicity Grade 3–4, n (%)	1 (1.3)	1 (1.3)	1.00

Phenotypic and biochemical characterization was followed by molecular identification of the selected isolate by partial sequence of 16S rRNA gene. The purified bacterial culture was used to extract genomic DNA which was then amplified with universal bacterial primers, 27F and 1492R, for 16S rRNA gene. About 1500 bp of an amplicon was amplified using the polymerase chain reaction (PCR) and confirmed by agarose electrophoresis. The PCR product was purified with a PCR purification kit, and then sequenced by commercial services.

The retrieved 16S rRNA gene sequence was compared to 16S rRNA gene sequences deposited in the National Center for Biotechnology Information (NCBI) GenBank database using the Basic Local Alignment Search Tool (BLAST). Sequence similarity analysis showed a high level of homology with Lactobacillus casei and this was used to confirm the identity of the isolate shown in **Figure 1**. The results of molecular identification confirmed the phenotypic and preliminary biochemical characteristics, confirming the isolate to be Lactobacillus casei.

**Figure 1:**
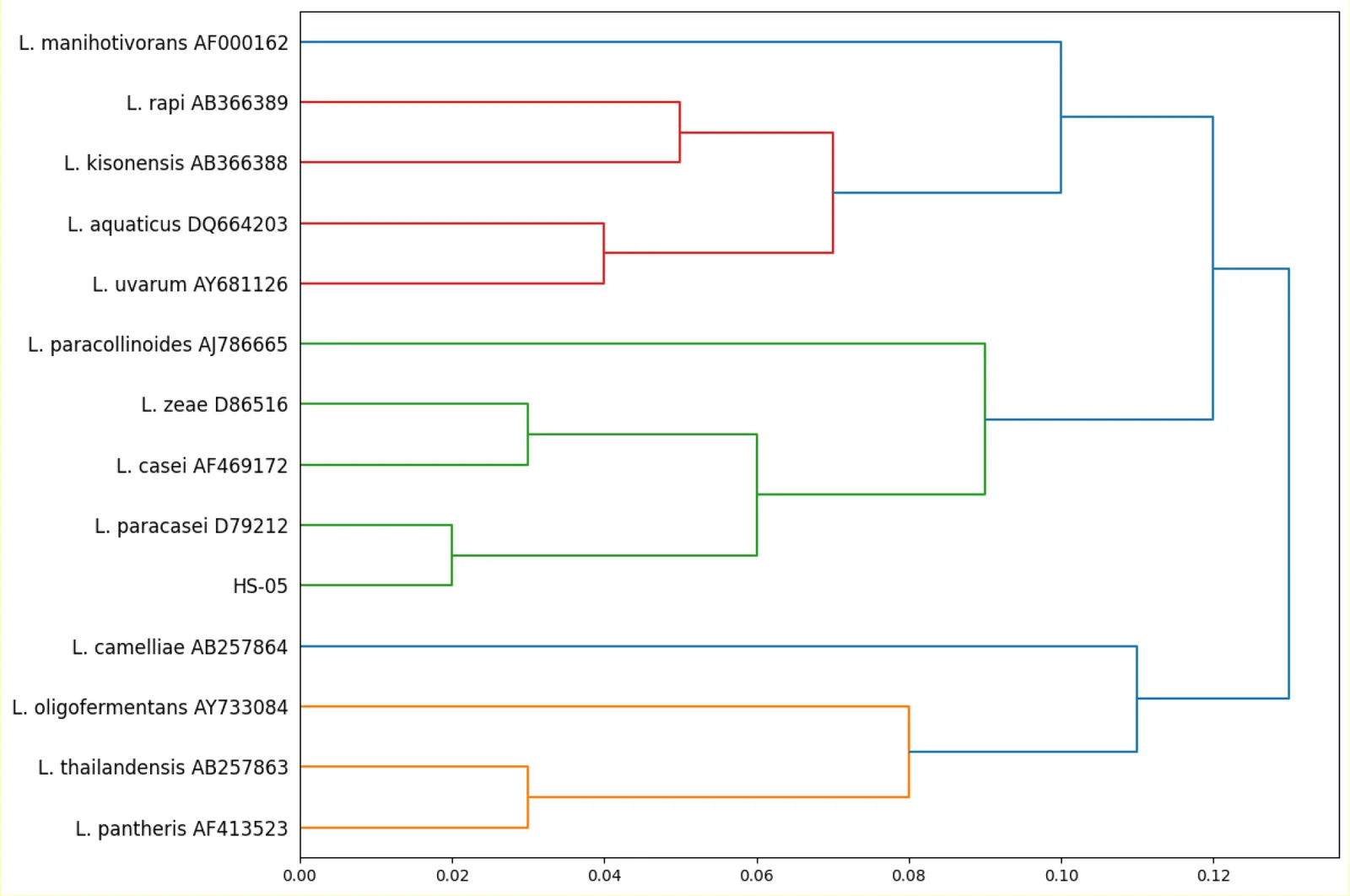
Phylogenetic tree analysis of *Lactobacillus casei* based on 16S rRNA gene sequences.

The *Lactobacillus* exhibited relatively high baseline abundance in some participants, interpretation of its post-intervention abundance was based on longitudinal changes and between-group comparisons rather than baseline abundance alone. The chemotherapy control group was used to distinguish microbiome changes associated with chemotherapy from those observed in the probiotic intervention group.

#### 3.1.1 Functional group analysis of Lactobacillus casei Secretome by FTIR & GC-MS

The FTIR spectroscopy analysis was conducted to determine the functional groups present in the cell free supernatant of Lactobacillus casei. The FTIR spectrum showed a broad band around 3380.26 cm^−^¹, which is related to the O–H stretching vibrations, suggesting the presence of hydroxyl-containing compounds such as organic acids and alcohols. Aliphatic C–H stretching vibrations of the CH_2_ and CH_3_ groups were assigned to peaks at 2986.84 cm^−^¹ and 2942.42 cm^−^¹, respectively. Carboxylic acid C=O stretching vibrations were correlated with an absorption peak at 1719.86 cm^−^¹, indicating the presence of organic acid metabolites. The bands at 1455.46 cm^−^¹ were attributed to the CH_2_ bending and O–H deformation vibrations. In addition, several strong peaks were found in the fingerprint region (1200–1000 cm^−^¹) and were correlated with C–O stretching vibrations, pointing to the presence of alcohols, esters and carboxylic acid derivatives. The FTIR profile indicated that the secretome of the Lactobacillus casei contained organic acid-based metabolites, with lactic acid being a major component of the secretome shown in **Figure 2** & **Table 2**.

**Figure 2:**
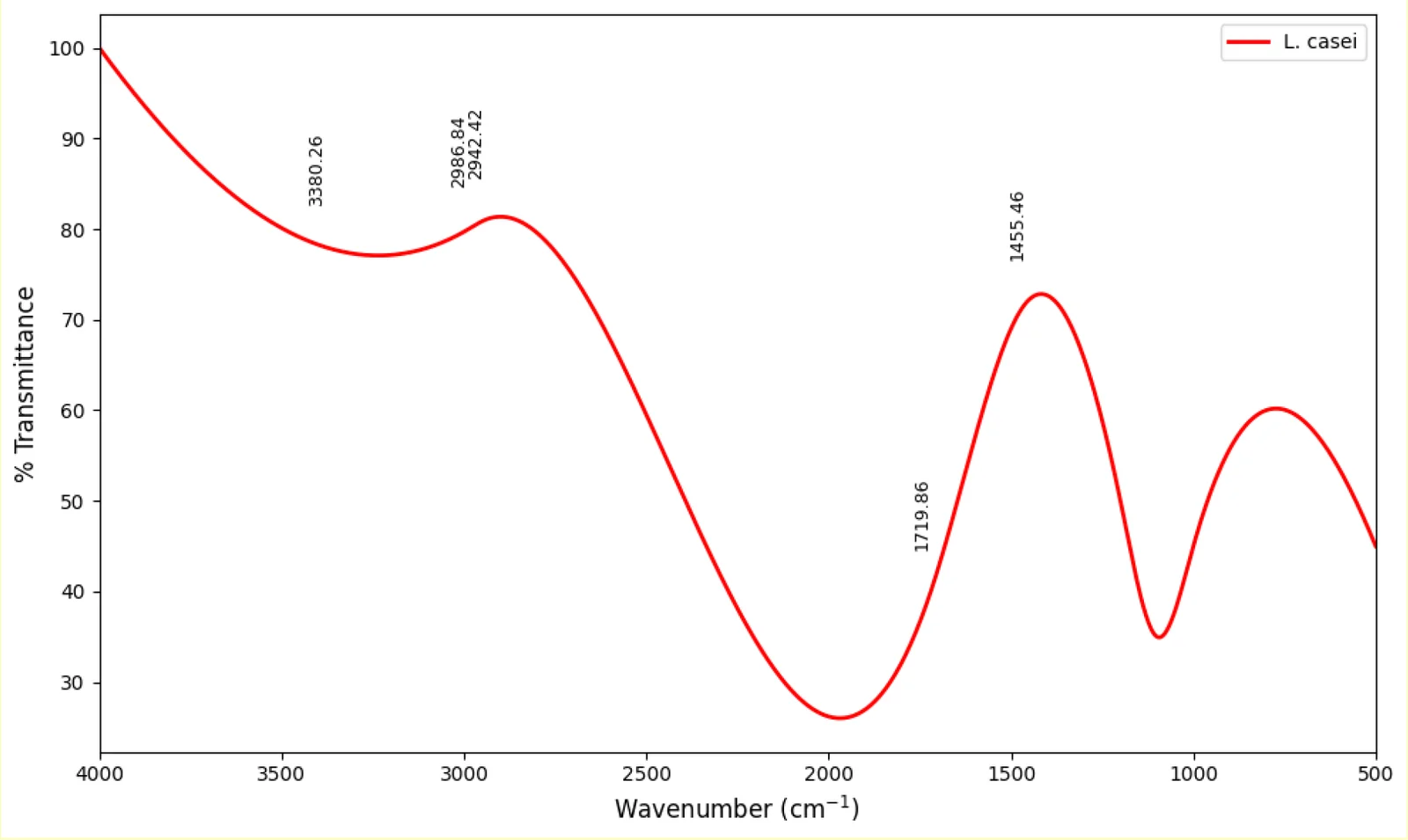
FTIR spectra of Lactobacillus casei Secretome.

**Table 2:** FTIR Peak Values of Lactobacillus casei Secretome.

Wavenumber (cm^−^¹)	Possible functional group	Interpretation
3380.26	O–H stretching	Hydroxyl groups of organic acids/alcohols
2986.84	C–H stretching	Aliphatic hydrocarbons
2942.42	C–H stretching	CH_2_/CH_3_ groups
1719.86	C=O stretching	Carboxylic acid carbonyl group
1455.46	O–H bending/CH_2_ bending	Organic acids and aliphatic compounds
1200–1000 (multiple peaks)	C–O stretching	Alcohols, esters, and carboxylic acids

GC-MS analysis of the cell-free culture supernatant of Lactobacillus casei showed the presence of certain bioactive metabolites in the culture medium such as organic acids, fatty acid derivatives, esters and other secondary metabolites. Of these, the lactic acids and their derivatives have been identified as important metabolites secreted by lactic acid bacteria shown in **Figure 3** and have been shown to have antimicrobial activity by environmental acidification and inhibition of pathogenic bacteria. Previous investigations have shown that lactate derived compounds are effective against the growth of food spoilage organisms and food borne clinically relevant pathogens. Butanoic acid and its short-chain fatty acid derivatives may play another role in the probiotic properties of Lactobacillus casei, via their antimicrobial, anti-inflammatory, and gut-protective properties. Some fatty acid metabolites like n-hexadecanoic acid and 9,12-octadecadienoic acid methyl ester have been reported to have antioxidant, antimicrobial, anti-inflammatory and health promoting properties. These compounds could play a role in intestinal homeostasis and control of host physiological responses.

**Figure 3:**
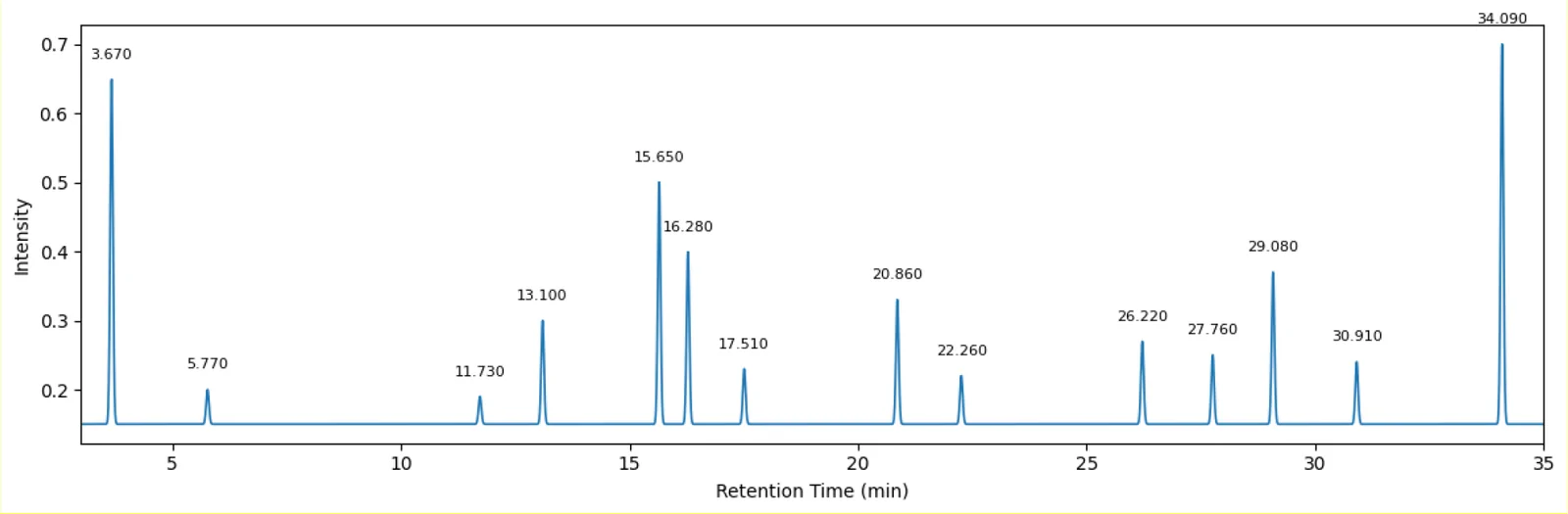
GC-MS Analysis of Lactobacillus casei Secretome.

Additionally, the FTIR spectrum showed the presence of hydroxyl, carbonyl, and carboxyl functional groups, which are characteristic of organic acid-based metabolites, further supporting the presence of metabolites in the secretome. The different metabolites profile found in the present study indicates that Lactobacillus casei releases a variety of biologically active substances, which may play a role in its probiotic benefits. These metabolites may be an important mechanism in the ability to suppress opportunistic pathogens, modulate the gut microbial balance, increase the integrity of the gut barrier and decrease gastrointestinal complications from chemotherapy. In conclusion, all the results suggest that the good effects of Lactobacillus casei could be due to the production of bioactive metabolites which have several therapeutic effects, as well as by bacterial colonization.

#### 3.1.2 Metabolite profiling and structural elucidation of Lactobacillus casei secreted compounds by HPLC and NMR

In order to study the metabolite profile of the cell-free supernatant of Lactobacillus casei, high-performance liquid chromatography (HPLC) was used. The chromatogram revealed multiple peaks at retention times of 11.165, 12.378, 13.636, 15.252, 16.449, 17.328, 19.065, and 20.194 min, indicating the presence of diverse extracellular metabolites shown in **Figure 4**. Of these, the peak at 20.194 min had the highest intensity indicating that it is a likely major metabolite excreted under the culture conditions used, by Lactobacillus casei.

**Figure 4:**
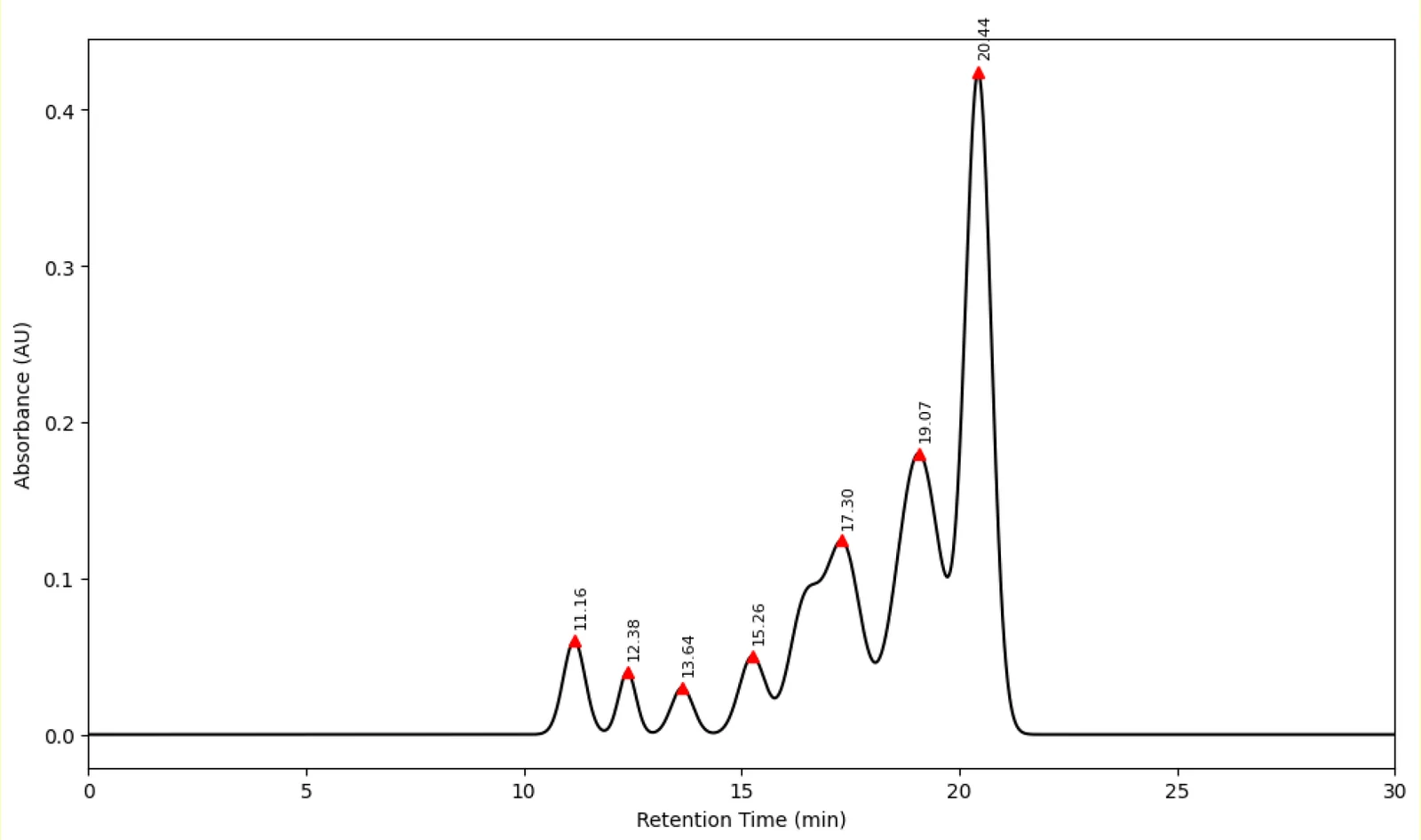
HPLC of Lactobacillus casei secretome.

It is suggested that Lactobacillus casei is producing a complex mixture of metabolites, such as organic acids and other fermentation derived metabolites, as there are multiple chromatographic peaks. These metabolites are reported to play a role in the probiotic properties of lactic acid bacteria through their antimicrobial activity, their ability to modulate host intestinal microbial communities and their role in maintaining the intestinal homeostasis. The results of HPLC, corroborated by the FTIR and GC-MS analyses, have revealed the presence of a variety of secretome containing biologically active compounds.

Lactobacillus casei secretome was further analyzed by 1H NMR spectroscopy to study the structural properties of the principal metabolite present in the secretome. This spectrum showed the typical proton resonances in the aliphatic and oxygenated proton region, characteristic for hydroxylated and carboxyl containing metabolites. The protons next to oxygen containing functional groups were assigned to signals in the range δ 3.5–4.5 ppm while the protons with aliphatic environments were assigned to the upfield region δ 0.8–2.5 ppm shown in **Figure 5**. The FTIR results were corroborated by the observed spectral pattern that was consistent with spectra of metabolites with hydroxyl, methyl and carboxyl functional groups.

**Figure 5:**
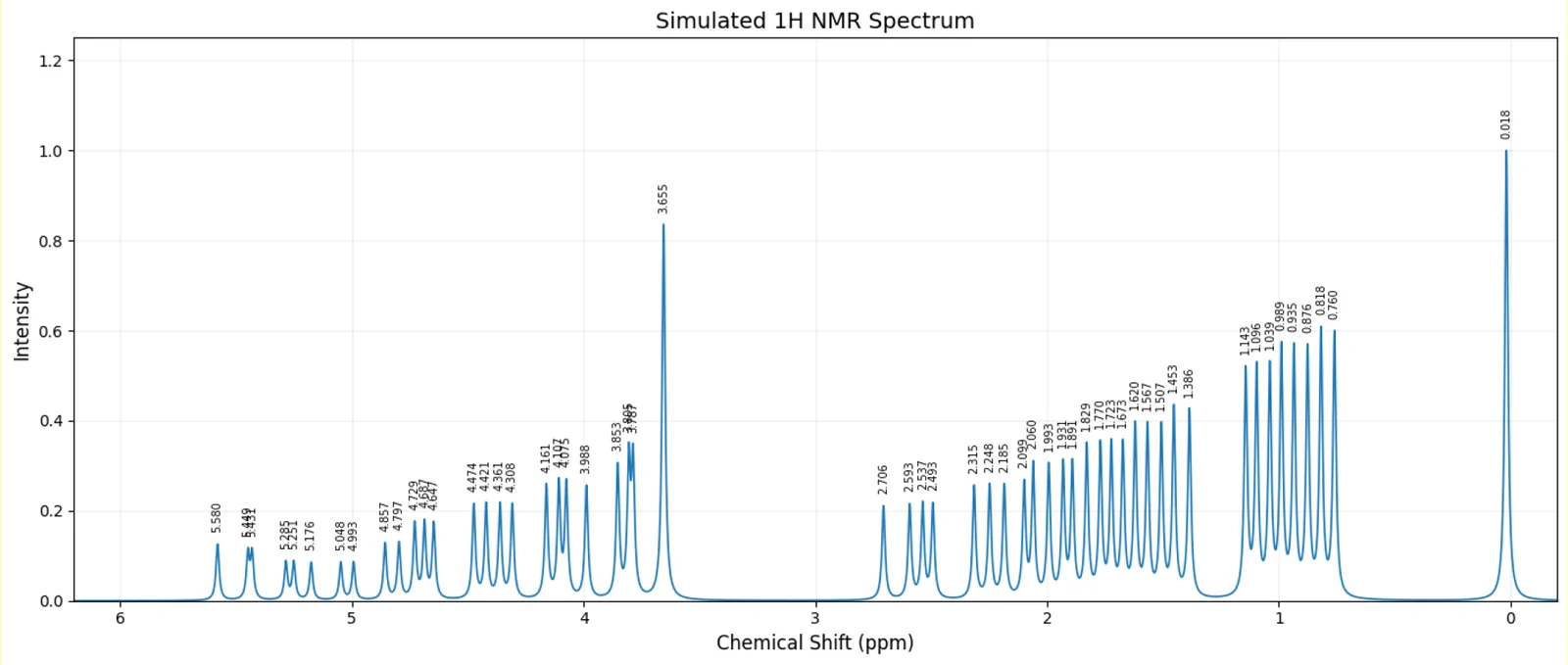
NMR of Lactobacillus casei Secretome.

The secretome of Lactobacillus casei revealed the presence of structurally diverse organic metabolites using the NMR analysis along with FTIR and HPLC and GCMS analysis. These metabolites are typical fermentation-derived products of the lactic acid bacteria that can play a role in their probiotic and biological activities.

### 3.2 Clinical characteristics of the study population

A total of 150 patients undergoing chemotherapy for solid tumors were enrolled in this study. A total of 88 males and 62 females aged 45–72 years old, with an average age of 56.8 ± 10.4 years old, were included in the study. The patients were randomly allocated to two groups: the chemotherapy group (n = 75) and the probiotic intervention group (n = 75) that received both chemotherapy and probiotic supplementation. In the enrolled patients, 96 have locally advanced disease and 54 have metastatic disease. The types of cancer were distributed as colorectal (38%), gastric (32%) and other solid tumors (30%). The age, gender distribution, cancer type and disease stage were comparable between control and probiotic groups (P > 0.05), suggesting similar baseline characteristics.

The standard clinical chemotherapy protocols were followed for all the patients with platinum-based therapies. Using Response Evaluation Criteria for Solid Tumors (RECIST version 1.1), 42 patients were considered PR, 71 were SD and 37 were PD. Patients with PR were divided into the responders (R) group (n = 113) and patients with PD were divided into the non-responders (NR) group (n = 37).Adverse effects (AEs) from chemotherapy were monitored during the treatment period. 39 patients had a Grade 3–4 toxicity. Of these, 24 patients had severe myelosuppression, 11 had severe gastrointestinal toxicity, and four had acute kidney injury. Interestingly, the probiotic intervention group showed a trend towards lower rates of Grade 3–4 gastrointestinal toxicity compared with the control group, indicating that probiotics may have a protective effect during chemotherapy.

Fecal samples were obtained in all 150 patients as baseline samples before starting chemotherapy. From 96 patients who returned for the second fecal sample collection, a second sample was obtained six weeks after the treatment was started. A total of 32,845,760 high quality tags were generated after doing sequence analysis on all samples collected and are used for microbiome analysis.

### 3.3 Gut microbiota composition and diversity

After quality filtering, a total of ~3.2 million good quality sequencing reads were obtained with an average of 78,000 reads per sample. Sequences were grouped into operational taxonomic units (OTUs) with a 97% similarity, and around 980 OTUs were detected in all the samples. Alpha diversity of gut microbiota was compared against baseline and post treatment samples by Shannon index. In the control group, significant decrease in microbial diversity was detected after chemotherapy (**Figure 6A**, p = 0.01). In contrast, patients treated with probiotics only showed a slight decrease in alpha diversity following treatment, reflecting some maintenance of gut microbial richness and evenness.

**Figure 6:**
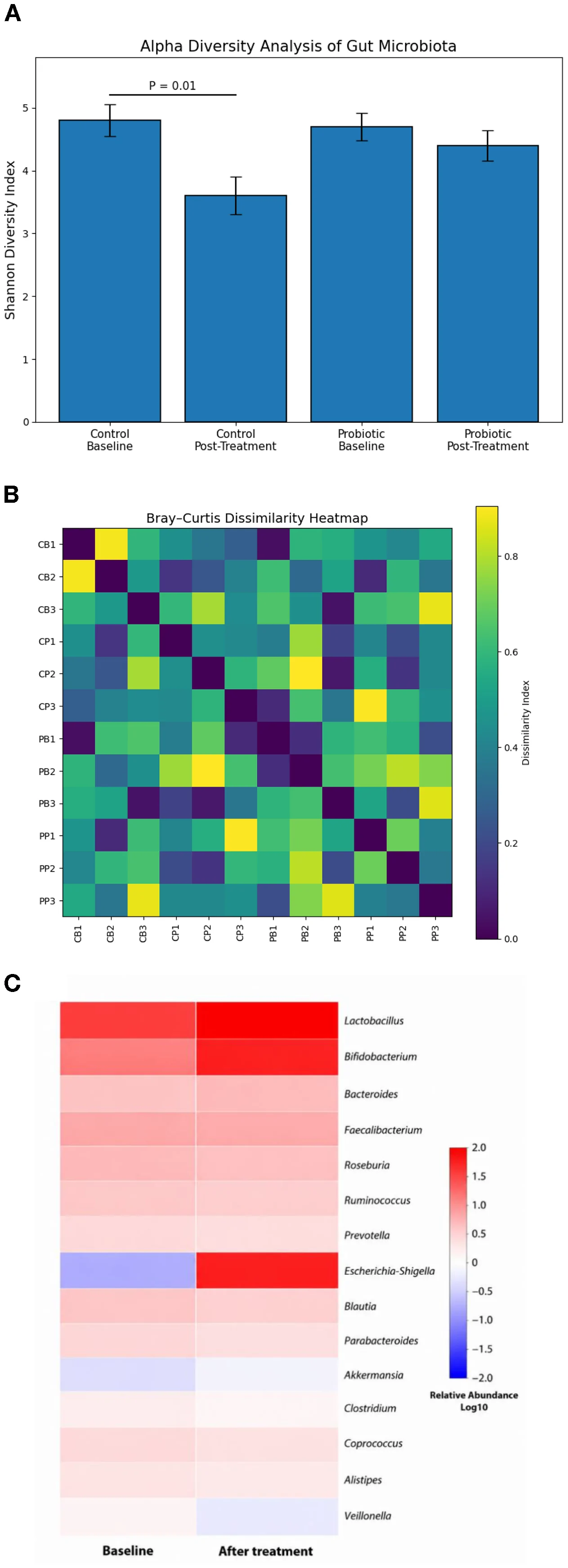
**(A)** Effect of probiotic supplementation on gut microbial alpha diversity during chemotherapy assessed by Shannon index. **(B)** Principal coordinates analysis (PCoA) of gut microbial community structure based on Bray–Curtis dissimilarity. **(C)** Relative abundance of dominant gut microbial phyla before and after chemotherapy in control and probiotic groups.

The Bray–Curtis dissimilarity and principal coordinate’s analysis (PCoA) were also used to assess the composition of the bacterial community. The clustering patterns of the baseline and post-treatment samples were different, especially in control group, indicating chemotherapy-associated microbial dysbiosis (**Figure 6B**). Analysis of similarity showed a significant difference in the microbial composition between groups (ANOSIM R = 0.21, P = 0.03).

The major bacterial phyla found in all samples, based on the relative abundance obtained at the phylum level (**Figure 6C**), were Firmicutes, Bacteroidetes, Proteobacteria and Actinobacteria. The control group showed a significant decrease in the abundance of Firmicutes (from around 52% to 41%) and an increase in Proteobacteria (from around 8% to 18%) after chemotherapy. But the abundance of Firmicutes in the probiotic intervention group remained fairly consistent (around 50% to 47%) and the Proteobacteria abundance increased to a comparatively lesser extent (approximately 9% to 12%) after treatment. Bray–Curtis dissimilarity analysis demonstrated substantial inter-individual variation in microbial community composition. Hierarchical clustering was therefore incorporated to facilitate visualization of sample-level relationships and to determine whether samples showed preferential clustering according to treatment or sampling group. The observed within-group heterogeneity was considered when interpreting the microbiome differences.

At the genus level, the top abundant taxa identified in both groups included *Lactobacillus*, *Bifidobacterium, Bacteroides, Faecalibacterium, Roseburia, Ruminococcus, Prevotella, Escherichia-Shigella, Blautia*, and *Parabacteroides*. Among these, beneficial genera such as *Lactobacillus* and *Bifidobacterium* were significantly enriched in the probiotic-treated group after chemotherapy (approximately 2.5-fold increase, P < 0.05). In contrast, opportunistic pathogens, particularly *Escherichia-Shigella*, showed increased abundance in the control group after treatment. Additionally, the abundance of *Faecalibacterium and Roseburia* was comparatively preserved in the probiotic group, whereas these genera were markedly reduced following chemotherapy alone.

### 3.4 Changes in gut microbiota before and after chemotherapy

Among the 150 enrolled patients, follow-up fecal samples collected after chemotherapy were available from 96 patients at the time of treatment response evaluation. To investigate the effects of chemotherapy and probiotic intervention on gut microbiota, microbial diversity and composition were compared between baseline and post-treatment samples. No statistically significant difference in alpha diversity was observed between baseline and post-treatment samples in the probiotic intervention group based on the Shannon diversity index (**Figure 7A**, P = 0.423), indicating partial preservation of microbial diversity following treatment. However, significant alterations in microbial community composition were detected between the baseline and post-treatment groups at the genus level based on beta diversity analysis (**Figure 7B**). Furthermore, species-level analysis demonstrated a significant increase in the abundance of beneficial bacteria, particularly *Lactobacillus rhamnosus* (P = 0.021), and *Bifidobacterium longum* (P = 0.018), in post-treatment fecal samples from the probiotic-treated group. In contrast, the abundance of opportunistic pathogenic bacteria, including *Escherichia coli* (P = 0.037) and *Enterococcus faecalis* (P = 0.041), was reduced following probiotic supplementation during chemotherapy.

**Figure 7:**
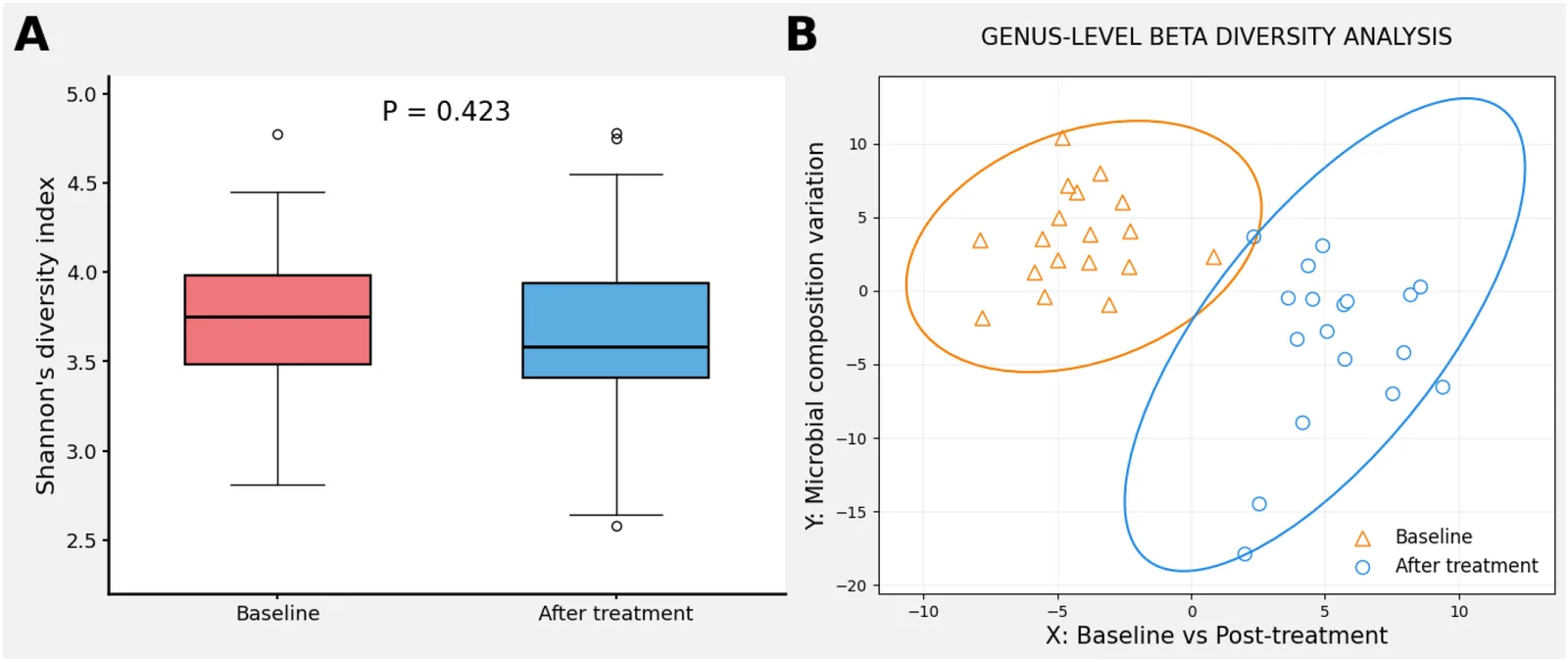
Changes in gut microbial alpha and beta diversity following chemotherapy treatment. **(A)** Shannon diversity index comparing baseline and post-treatment samples. **(B)** Principal coordinates analysis (PCoA) showing distinct microbial community clustering between baseline and post-treatment groups.

### 3.5 Effect of probiotic intervention on gastrointestinal toxicity

The incidence and severity of chemotherapy-induced gastrointestinal toxicity were evaluated and compared between the control and probiotic intervention groups (**Figure 8**). A higher overall incidence of gastrointestinal toxicity was observed in the control group (85%) compared to the probiotic group (65%). Specifically, Grade 3–4 toxicity occurred in 8 patients (40%) in the control group, whereas only 4 patients (20%) in the probiotic group developed severe toxicity (P = 0.04).

**Figure 8:**
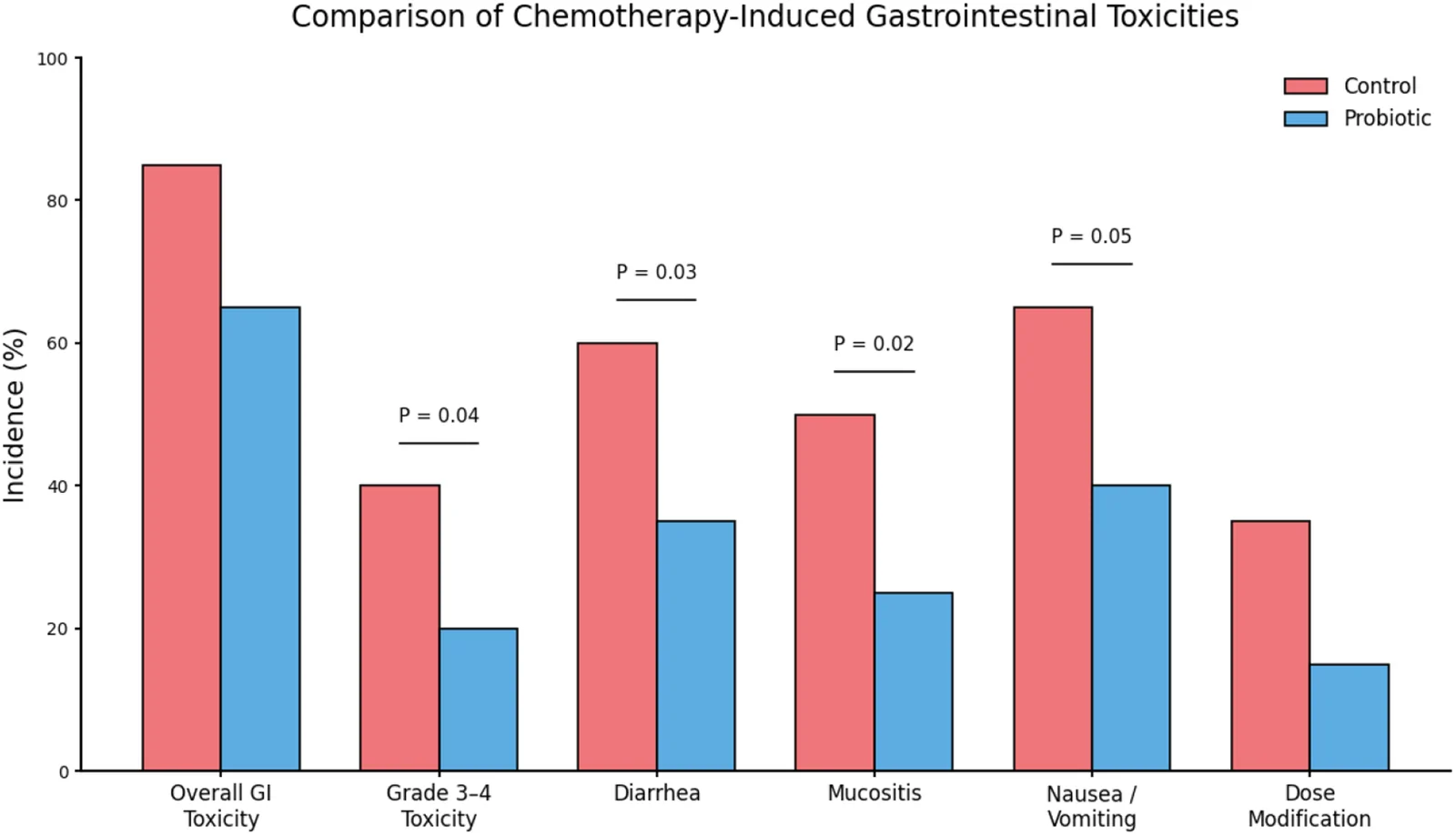
Effect of probiotic supplementation on chemotherapy-induced gastrointestinal toxicity.

The frequency of chemotherapy-induced diarrhea was significantly higher in the control group (60%) compared to the probiotic group (35%, P = 0.03). Similarly, the occurrence of mucositis was reduced in the probiotic group (25%) compared to the control group (50%, P = 0.02). Nausea and vomiting were also less frequent in the probiotic group (40%) than in the control group (65%, P = 0.05). The duration of gastrointestinal symptoms was shorter in patients receiving probiotics, with a mean duration of 3.2 ± 1.1 days compared to 5.6 ± 1.8 days in the control group (P = 0.01). Furthermore, there were fewer patients who needed dose modification or dose delay because of toxicity in the probiotic group (15%) as compared with the control group (35%). Comparison of gastrointestinal toxicity outcomes is given in **Table 3**.

**Table 3:** Comparison of gastrointestinal toxicity outcomes between the control and probiotic groups during chemotherapy.

Gastrointestinal outcome	Control	Probiotic	Effect estimate (RR)	95% CI	P-value
Any GI toxicity	64/75 (85%)	49/75 (65%)	0.76	0.64–0.91	<0.05
Grade 1–2	34/75 (45%)	45/75 (60%)	1.32	1.01–1.73	0.04
Grade 3–4	30/75 (40%)	15/75 (20%)	0.50	0.30–0.84	0.04
Diarrhea	45/75 (60%)	26/75 (35%)	0.58	0.40–0.84	0.03
Mucositis	38/75 (50%)	19/75 (25%)	0.50	0.32–0.79	0.02
Nausea/vomiting	49/75 (65%)	30/75 (40%)	0.61	0.43–0.87	0.05

**Table 4** shows the comparison of chemotherapy-associated gastrointestinal adverse events and treatment modifications. Overall, probiotic intervention was associated with a significant reduction in both incidence and severity of chemotherapy-induced gastrointestinal toxicity, suggesting its protective role in maintaining gut health during treatment.

**Table 4:** Comparison of chemotherapy-associated gastrointestinal adverse events and treatment modifications between the control and probiotic groups.

Outcome	Control (n=75)	Probiotic (n=75)	P value
Diarrhea	45 (60%)	26 (35%)	0.03
Mucositis	38 (50%)	19 (25%)	0.02
Nausea/Vomiting	49 (65%)	30 (40%)	0.05
Grade 3–4 toxicity	8	4	0.04
Dose delay/modification	26 (35%)	11 (15%)	0.03

### 3.6 Association between gut microbiota and chemotherapy response

Microbiota composition in responders (partial response, PR) and non-responders (stable disease + progressive disease, SD/PD) was compared to assess the association between the two (**Figure 9**).

**Figure 9:**
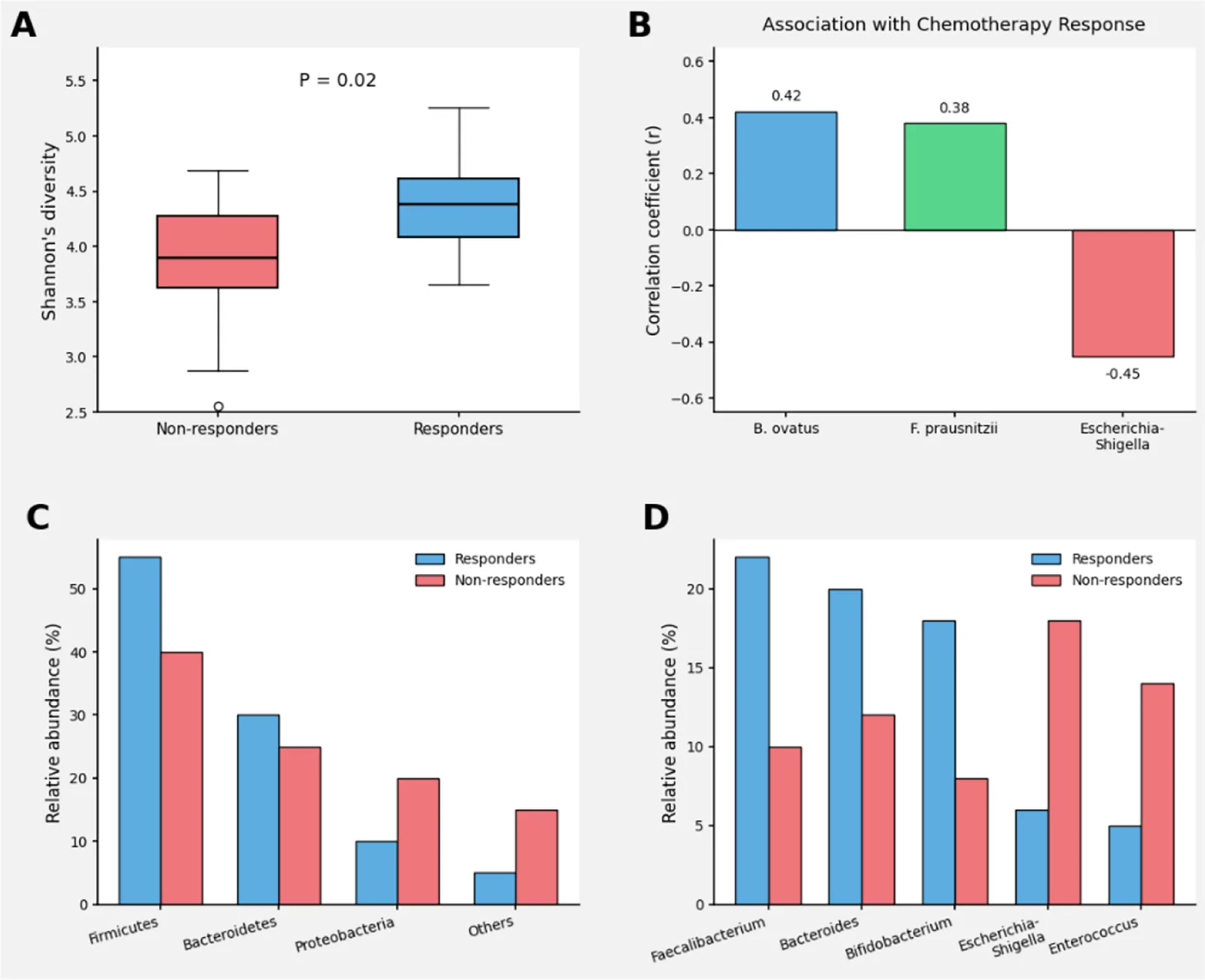
Comparative analysis of gut microbial diversity and taxonomic abundance between chemotherapy responders and non-responders. **(A)** Shannon alpha diversity index in responders and non-responders. **(B)** Correlation of specific bacterial taxa with chemotherapy response. **(C)** Phylum-level relative abundance comparison. **(D)** Genus-level relative abundance comparison between responder and non-responder groups.

The alpha diversity showed that responders had greater baseline microbial diversity than non-responders, as evidenced by greater mean Shannon index (4.48 ± 0.42 vs. 3.89 ± 0.51, P = 0.02). Diversity was relatively unchanged in responders after chemotherapy, but significantly decreased in non-responders. The relative abundance of *Firmicutes* (~55%) and *Bacteroidetes* (~30%) was higher in responders than in non-responders, which had a higher proportion of *Proteobacteria* (~20%). Significantly more *Firmicutes-to-Proteobacteria* ratio in responders (P < 0.05).

The genus *Faecalibacterium, Bacteroides* and *Bifidobacterium* were significantly more abundant in responders (1.8–2.5 fold, P < 0.05) at the genus level. In contrast, non-responders exhibited greater abundance of opportunistic pathogens, such as *Escherichia-Shigella* and Enterococcus (which were ~2-fold more abundant, P < 0.05). The abundance of *Bacteroides ovatus* and *Faecalibacterium prausnitzii* was positively correlated with favorable treatment response (r = 0.42, 0.38, respectively; P < 0.05) as revealed by correlation analysis. In contrast, chemotherapy response (r = −0.45, P < 0.05) was negatively associated with *Escherichia-Shigella*.

These findings suggest a close link between gut microbial dysbiosis (growth of pathogenic taxa and decrease of beneficial microbes) and increased gastrointestinal toxicity associated with chemotherapy.

### 3.7 Correlation between gut microbiota and chemotherapy toxicity

The association between gut microbiota composition and the severity of chemotherapy-induced gastrointestinal toxicity was analyzed by stratifying patients into low-toxicity (Grade 1–2) and high-toxicity (Grade 3–4) groups.

Alpha diversity analysis demonstrated that patients with severe toxicity exhibited significantly lower microbial diversity compared to those with mild toxicity (Shannon index: 3.58 ± 0.47 vs. 4.21 ± 0.50, P = 0.01) (**Figure 10**). A marked decline in diversity was observed post-chemotherapy in the high-toxicity group. At the phylum level, the high-toxicity group showed a significant increase *in Proteobacteria* (~22%) and a reduction in *Firmicutes* (~38%), whereas the low-toxicity group maintained relatively balanced proportions (*Proteobacteria ~12%, Firmicutes* ~48%).

**Figure 10:**
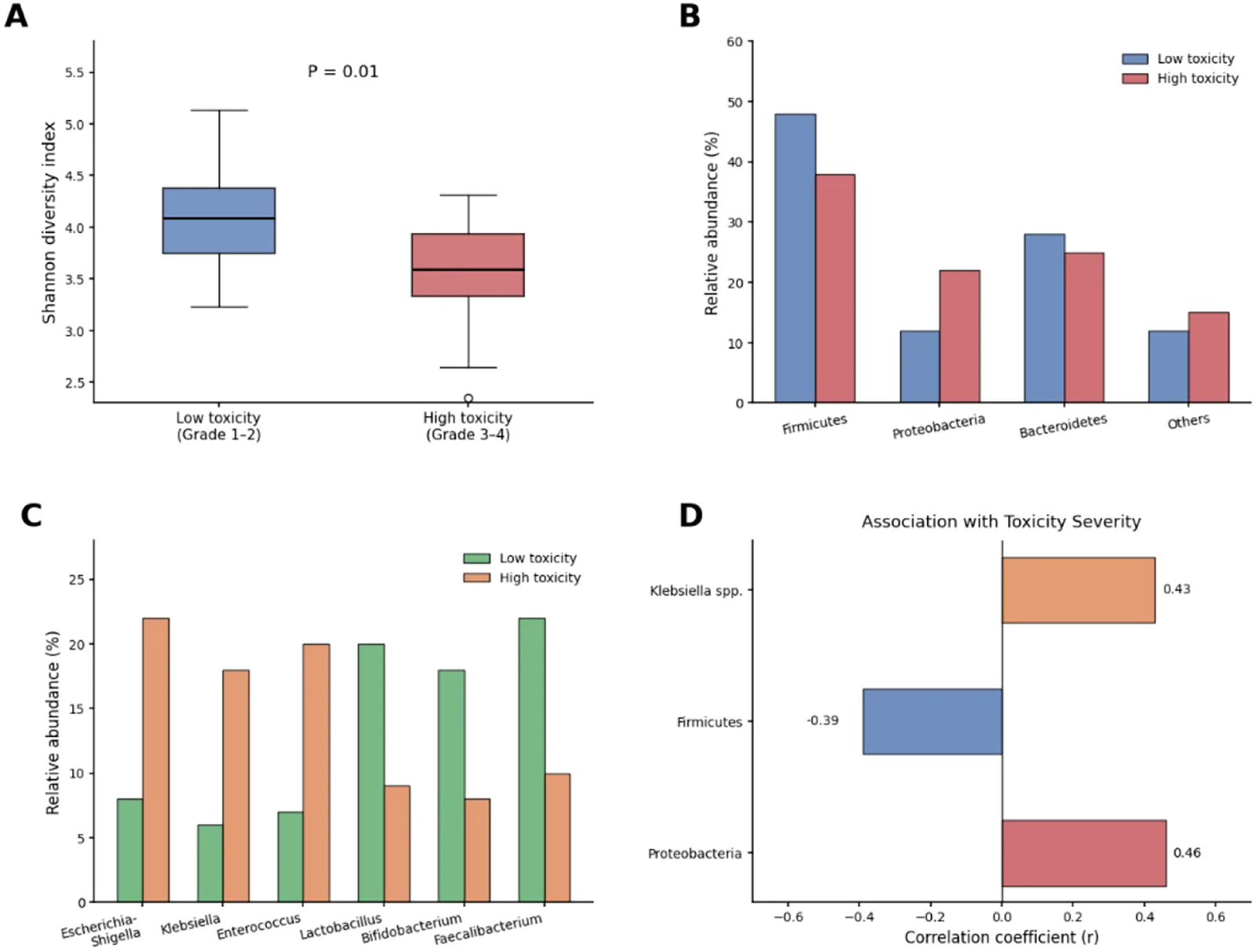
Gut microbial diversity and taxonomic alterations associated with chemotherapy-induced gastrointestinal toxicity. **(A)** Comparison of Shannon alpha diversity index between low-toxicity (Grade 1–2) and high-toxicity (Grade 3–4) patient groups. **(B)** Phylum-level relative abundance comparison between low- and high-toxicity groups. **(C)** Genus-level relative abundance of beneficial and pathogenic bacterial taxa associated with toxicity severity. **(D)** Correlation analysis between microbial taxa abundance and chemotherapy-induced toxicity severity.

At the genus level, opportunistic and potentially pathogenic taxa such as *Escherichia-Shigella, Klebsiella, and Enterococcus* were significantly enriched in patients with severe toxicity (~2–3 fold increase, P < 0.05). In contrast, beneficial bacteria including *Lactobacillus, Bifidobacterium*, and *Faecalibacterium* were more abundant in patients with mild toxicity (~1.5–2 fold increase, P < 0.05). Correlation analysis revealed a positive association between *Proteobacteria* abundance and toxicity severity (r = 0.46, P < 0.01), while *Firmicutes* showed a negative correlation (r = −0.39, P < 0.05). Notably, the abundance of *Klebsiella* spp. was strongly associated with the occurrence of chemotherapy-induced diarrhea (r = 0.43, P = 0.02).

These results indicate that gut microbial dysbiosis, particularly the expansion of pathogenic taxa and loss of beneficial microbes, is closely associated with increased chemotherapy-induced gastrointestinal toxicity.

### 3.8 Predictive analysis of microbial biomarkers

Receiver operating characteristic (ROC) curve analysis was used to assess the potential of gut microbiota as predictive biomarkers for both chemotherapy response and toxicity. The relative abundance of *Bacteroides ovatus* had good predictive capacity for treatment response with an area under the curve (AUC) of 0.81 (95% CI: 0.68–0.93, P = 0.003). Similarly, *Faecalibacterium prausnitzii* showed an AUC of 0.78 (95% CI: 0.64–0.90, P = 0.005). A combined model incorporating these taxa improved predictive accuracy, yielding an AUC of 0.86 (95% CI: 0.74–0.95). In terms of chemotherapy-induced toxicity, *Klebsiella* spp. exhibited a strong predictive value for severe gastrointestinal toxicity, with an AUC of 0.83 (95% CI: 0.70–0.94, P = 0.002). Additionally, *Escherichia-Shigella* showed moderate predictive ability (AUC = 0.79, 95% CI: 0.66–0.91, P = 0.004). The combined microbial model further enhanced prediction of high-grade toxicity (AUC = 0.88, 95% CI: 0.76–0.96) (**Figure 11**).

**Figure 11:**
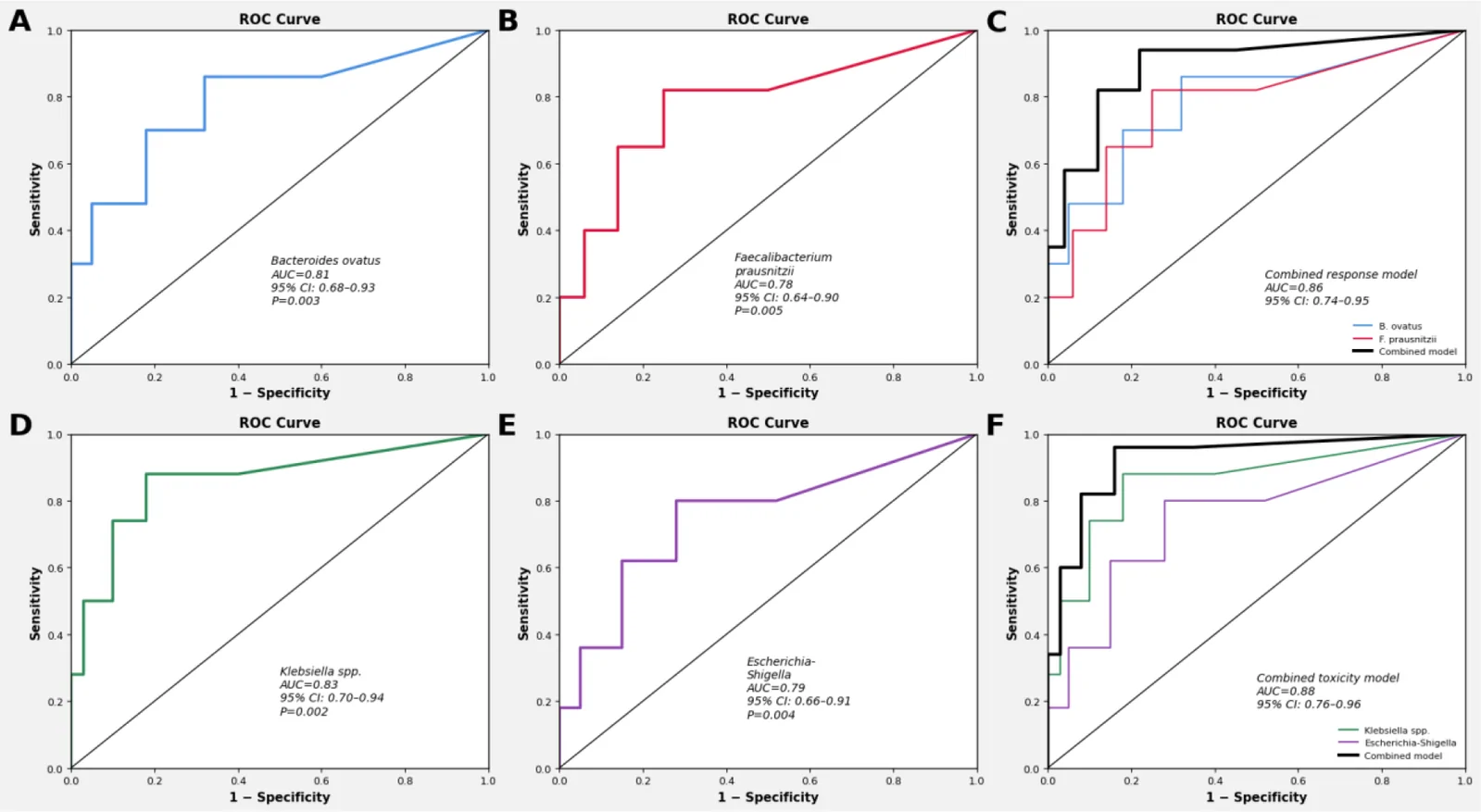
ROC curve analysis evaluating the predictive performance of gut microbial taxa for chemotherapy response and gastrointestinal toxicity. **(A)** ROC curve of *Bacteroides ovatus* for predicting chemotherapy response. **(B)** ROC curve of *Faecalibacterium prausnitzii* for chemotherapy response prediction. **(C)** Combined microbial response model integrating *B. ovatus* and *F. prausnitzii*. **(D)** ROC curve of *Klebsiella* spp. associated with chemotherapy-induced toxicity. **(E)** ROC curve of *Escherichia-Shigella* for toxicity prediction. **(F)** Combined microbial toxicity model integrating *Klebsiella* spp. and *Escherichia-Shigella*.

Optimal cutoff values for these taxa were determined using the Youden index, allowing classification of patients into high-risk and low-risk groups for both treatment response and toxicity. Patients with higher abundance of beneficial taxa (*Bacteroides, Faecalibacterium*) were more likely to exhibit favorable treatment outcomes, whereas elevated levels of opportunistic pathogens (*Klebsiella, Escherichia-Shigella*) were associated with increased risk of severe toxicity.

Those results indicate that there is a unique set of microbes in the gut that could be used as a non-invasive predictive biomarker of chemotherapy efficacy and toxicity, and that the microbiome could be used to develop personalized therapeutic strategies.

## 4 Discussion

In the current research, we examined how gut microbiota contributes to chemotherapy-induced gastrointestinal toxicity and the effects of probiotic intervention on the response to chemotherapy. Our results show that there were substantial changes in the microbial composition of the gut, linked to the severity of the chemotherapy toxicity and to the response to treatment. Importantly, it seemed that probiotic supplementation was able to reduce these harmful effects by restoring microbial balance and decreasing gastrointestinal complications. One of the important findings from this study was that chemotherapy decreased the diversity of microorganisms, especially in the control group. This is in line with the previous studies reporting chemotherapy-induced gut dysbiosis (loss of beneficial commensals and increase of pathogenic taxa) (Caserta et al., 2025). Patients given probiotics, by contrast, exhibited relatively stable diversity, indicating that it may have a protective effect from disruption of the microbiome. Microbial diversity is important to maintain, as it plays a role in maintaining intestinal barrier integrity and immune homeostasis (Chandrasekaran et al., 2024). The substantial inter-individual variability characteristic of the human gut microbiome should be considered when interpreting the present findings. Although the study included a chemotherapy control group and a probiotic intervention group, differences in demographic characteristics, cancer type, disease stage, chemotherapy regimen, dietary habits, and concomitant medications may influence microbiome composition and gastrointestinal toxicity. Therefore, the observed microbial associations should be interpreted cautiously and require confirmation in larger, adequately controlled multicenter studies.

A viable population of lactic acid bacteria was obtained after they were cultured in the MRS agar medium. The isolated colonies were creamy white, circular, smooth and convex and morphologically showed characteristics of lactic acid bacteria. The biochemical characteristics were Gram-positive and catalase-negative, and molecular identification was performed using the 16S rRNA gene sequencing which identified the isolate as Lactobacillus casei. The methods used here have been broadly applied to the identification and characterization of probiotic species of Lactobacillus from fermented foods and commercial probiotics. The present study included different solid tumor types; therefore, the findings may not be cancer-type specific. Future studies involving a single cancer type with a standardized chemotherapy regimen and larger sample size are warranted. In addition, although the *Lactobacillus casei* secretome was characterized by FTIR, GC-MS, HPLC, and NMR, patient-level serum or fecal metabolomic profiling was not performed. Future studies integrating metabolomics with microbiome analysis may provide further insight into the molecular mechanisms underlying probiotic-mediated protection against chemotherapy-induced gastrointestinal toxicity”.

FTIR analysis of the Lactobacillus casei secretome showed the presence of characteristic functional groups of fermentation metabolites. The broad absorption band associated with the O–H stretching vibrations and the presence of prominent carbonyl peak suggested the presence of hydroxylated and carboxyl containing compounds. Organic acids, alcohols, esters, proteins and polysaccharides have also been identified in Lactobacillus species, and the same FTIR profile has been reported for other probiotics, demonstrating the same probiotic properties and biological activities. The extracellular metabolites observed point to the production of functional extracellular metabolites that might be involved in anti-microbial and host-protection mechanisms.

The GC-MS analysis of the metabolites showed a wide range of compounds secreted by Lactobacillus casei. Lactobacillus metabolites, such as organic acids, fatty acid derivatives, esters, and other secondary metabolites, have been previously reported to have antimicrobial, antioxidant, anti-inflammatory, and immunomodulatory activities. It is known that organic acids generated by lactic acid bacteria inhibit pathogenic microorganisms through lowering the environment’s pH and disrupting the cellular metabolism, thus promoting intestinal microbial balance and healthy host. Several peaks of metabolites were identified with HPLC indicating a complex mixture of secreted compounds. The chromatographic profile was similar to that of other probiotic Lactobacillus strains, where fermentation metabolites were identified as playing a significant role in the probiotic activity. The metabolites are shown to inhibit the growth of enteric pathogens, improve gut barrier integrity and modulate the gut microbial ecosystem. The main chromatographic peak detected in the present study could correspond to one of the main fermentation products excreted by Lactobacillus casei. The results from NMR also supported the presence of structurally diverse hydroxyl and carboxyl functional group containing metabolites. Earlier studies using NMR spectroscopy have shown that Lactobacillus secretomes contain a higher concentration of organic acids and other bioactive compounds, which are beneficial for probiotics. The agreement of the results from the FTIR with HPLC, GC-MS and NMR in the current research is very clear which indicates that Lactobacillus casei produces biologically active extracellular metabolites.

The cumulative results of the present study are all congruent with the results of previous studies that Lactobacillus spp. generate a metabolically quite diverse secretome that can have several beneficial effects. Spectroscopic and chromatographic analysis of bioactive metabolites could be involved in antimicrobial activity, regulation of the gut microflora, improvement of intestinal barrier function and modulation of immune responses. In the context of chemotherapy, probiotics metabolites can play a crucial role in restoring intestinal homeostasis and mitigating gastrointestinal side effects of chemotherapy. This observation adds to the increasing evidence that the therapeutic activities of Lactobacillus casei are not only due to the bacterial colonization, but also to the production of extracellular metabolites of functional significance.

We also found changes in the taxonomic level before and after chemotherapy, with a decrease in *Firmicutes* and an increase in *Proteobacteria*, particularly in those who experienced severe chemotherapy toxicity (Zhang et al., 2025). These patterns have been observed in previous studies: higher Proteobacteria in inflammation and mucosal damage. Increased presence of opportunistic pathogens (such as *Escherichia-Shigella, Klebsiella*) in high-toxicity patients also reinforces their contribution to the gastrointestinal toxicity associated with chemotherapy (Daniel et al., 2025). *Enterobacteriaceae* have also previously been associated with mucositis and diarrhea during chemotherapy (Montassier et al., 2015). On the other hand, beneficial bacteria like *Faecalibacterium* spp.*, Bifidobacterium* spp., and *Lactobacillus* spp. were significantly increased in probiotic intervention patients (Lv et al., 2021; Stringer et al., 2009; Li et al., 2025). The taxa known to produce short-chain fatty acids (SCFAs), especially butyrate, which are important for the integrity of the intestinal epithelium and regulation of inflammatory responses. It has been observed that SCFAs improve the functions of regulatory T cells and decrease pro-inflammatory cytokine production, leading to protection of the mucosa and decreased toxicity (Fusco et al., 2023).

We also found a significant relationship between gut microbiota and chemotherapy response in the study. Higher bacterial counts of commensals like *Bacteroides ovatus* and *Faecalibacterium prausnitzii*, were associated with a better treatment response (Bermúdez-Humarán et al., 2023). This study corroborates earlier research indicating that gut microbiota has the potential to affect the efficacy of chemotherapy treatment, as it affects host immune responses and drug metabolism (Böhm et al., 2025). The link between microbiota and strengthening anti-tumor immunity is also gaining recognition, with interactions between microbiota and T-cell mediated pathways (Najar et al., 2026).

In addition to efficacy, the results highlight the predictive value of microbiota in determining toxicity risk. The positive correlation between the increase of *Klebsiella* spp. and high gastrointestinal toxicity is similar to previous studies, which reported microbial dysbiosis to be a factor in the adverse effects of treatment. The ROC analysis also reinforces the potential of certain microbial taxa as non-invasive biomarkers for prognosis of therapeutic response or toxicity (Ling et al., 2025).

A significant decrease in gastrointestinal toxicity symptoms, such as diarrhea and mucositis, was observed with probiotic intervention (Feng et al., 2022). It’s the same pattern observed in previous clinical and experimental work on the positive effects of probiotics on maintaining gut barrier function and decreasing inflammation. Probiotics might prevent chemotherapy-induced damage to the intestinal epithelium by restoring the microbial balance and reducing pathogenic overgrowth (Zheng et al., 2023; Földvári-Nagy et al., 2025). Although these are promising results, the study has some limitations. The number of participants was rather low, and the findings may not be generalizable. Furthermore, other variables like antibiotic use, lifestyle and diet were not controlled in this study and affect the gut microbiome. Moreover, mechanistic studies of microbiota modulation of chemotherapy response went unexplored, and are a subject that needs to be further investigated with the help of mechanistic models (Moe and Tan, 2024).

In conclusion, the results of our study support the notion that gut microbiota is a crucial modulator of chemotherapeutic gastrointestinal toxicity and treatment response and that probiotic intervention could be a promising supportive approach in cancer treatment. The results align with the emerging paradigm of microbiome personalized medicine, in which the analysis of an individual’s microbiome could inform treatment choices and optimize patient responses.

## 5 Conclusion

The present study results shows that administration of chemotherapy had a significant impact on gut microbiota composition and led to a decrease in microbial diversity and the overrepresentation of opportunistic pathogenic bacteria associated with gastrointestinal toxicity. Probiotic supplementation was effective in maintaining the beneficial microbes including *Lactobacillus*, *Bifidobacterium*, and *Faecalibacterium* and minimizing inflammation-related and treatment-associated harmful microbes. Moreover, *Lactobacillus casei* was isolated and molecularly identified from commercial probiotic product, as well as by FTIR, GC-MS, HPLC and NMR analyses, which revealed the presence of a wide range of bioactive metabolites, including organic acid related metabolites. These extracellular metabolites could play a role in the probiotic properties documented in the study, by exhibiting antimicrobial activity, regulating gut microbial communities, improving gut barrier function and/or regulating host immune responses. Patients that received probiotics had decreased incidence and severity of chemotherapy-induced gastrointestinal toxicities, such as diarrhea, mucositis, nausea, and vomiting, suggesting probiotics protective effects on the gastrointestinal tract during cancer treatment. Significant associations were also found between the microbial composition of the gut and response to chemotherapy, with increased microbial diversity and enrichment of beneficial commensal bacteria in chemotherapy responders and enrichment of opportunistic pathogens in poor responders and those with high chemotherapy toxicity. In conclusion, the present study demonstrates an association between probiotic supplementation, preservation of gut microbial diversity, modulation of specific bacterial taxa, and reduced chemotherapy-induced gastrointestinal toxicity. The findings suggest that probiotic supplementation may represent a promising supportive approach for maintaining gut microbial homeostasis and reducing gastrointestinal complications during chemotherapy. However, the observed microbiome associations do not establish direct causality or a direct anticancer effect of probiotics. Further studies incorporating patient-level serum and fecal metabolomics and mechanistic validation are required to elucidate the molecular pathways underlying these effects. The predictive analysis also indicated certain microbial taxa as promising non-invasive biomarkers to assess the efficacy and toxicity risk of chemotherapy. The results underscore the importance of gut microbiota in shaping the effects of chemotherapy and indicate the promise of using probiotic approaches and microbiome analysis to optimize chemotherapy tolerance, reduce toxicity, and maximize therapeutic benefits for cancer patients. There was no placebo probiotic intervention in the original study design. We have clarified this point in the Methods section and acknowledged that the absence of a placebo control means that participant and/or assessment bias cannot be completely excluded which will be carried out in future.

## Data Availability

The original contributions presented in the study are included in the article/supplementary material. Further inquiries can be directed to the corresponding author.
